# Study of TLR3, TLR4 and TLR9 in breast carcinomas and their association with metastasis

**DOI:** 10.1186/1471-2407-10-665

**Published:** 2010-12-03

**Authors:** Salomé González-Reyes, Laura Marín, Lucía González, Luis O González, José M del Casar, Maria L Lamelas, José M González-Quintana, Francisco J Vizoso

**Affiliations:** 1 Unidad de Investigación, Fundación Hospital de Jove, Gijón, Spain; 2 Servicio de Anatomía Patológica, Fundación Hospital de Jove, Gijón, Spain; 3 Servicio de Cirugía General, Fundación Hospital de Jove, Gijón, Spain

## Abstract

**Background:**

Toll-like receptors (TLRs) have garnered an extraordinary amount of interest in cancer research due to their role in tumor progression. By activating the production of several biological factors, TLRs induce type I interferons and other cytokines, which drive an inflammatory response and activate the adaptive immune system. The aim of this study was to investigate the expression and clinical relevance of TLR3, 4 and 9 in breast cancer.

**Methods:**

The expression levels of TLR3, TLR4 and TLR9 were analyzed on tumors from 74 patients with breast cancer. The analysis was performed by immunohistochemistry.

**Results:**

Samples of carcinomas with recurrence exhibited a significant increase in the mRNA levels of TLR3, TLR4 and TLR9. Tumors showed high expression of TLRs expression levels by cancer cells, especially TLR4 and 9. Nevertheless, a significant percentage of tumors also showed TLR4 expression by mononuclear inflammatory cells (21.6%) and TLR9 expression by fibroblast-like cells (57.5%). Tumors with high TLR3 expression by tumor cell or with high TLR4 expression by mononuclear inflammatory cells were significantly associated with higher probability of metastasis. However, tumours with high TLR9 expression by fibroblast-like cells were associated with low probability of metastasis.

**Conclusions:**

The expression levels of TLR3, TLR4 and TLR9 have clinical interest as indicators of tumor aggressiveness in breast cancer. TLRs may represent therapeutic targets in breast cancer.

## Background

Breast cancer remains a major cause of death in women in the developed world. One in nine women will suffer from breast cancer during her life [1]. Although clinical signs of disseminated disease occur in fewer than 10% of women at the time of diagnosis, the disease relapses in the form of metastasis within 5 years of surgery in about half of apparently localized tumours. It is difficult to predict the occurrence of distant metastases since breast cancer is a heterogeneous disease encompassing complex pathologic entities. For all of these reasons, new prognostic factors are essential to improving the classic risk classification in breast cancer.

Inflammation and cancer are related [2-8]. It is well known that persistent inflammatory conditions can induce cancer formation. This is in part, because cytokines and chemokines play a crucial role promoting angiogenesis, metastasis, and subversion of adaptive immunity[9].

Toll-like receptors (TLRs) are considered a link between innate (non-specific) and adaptive (specific) immunity and TLRs contribute to the immune system's capacity to efficiently combat pathogens [10]. This is done by means of the induction of signaling cascades resulting in the induction of type I interferons (IFNs) and other cytokines. The result of this process leads to an inflammatory response and activates the adaptative immune system [11]. TLRs also enable immune cells to discriminate between self and nonself antigens [12]. As molecular sensors, TLRs detect pathogen-derived products and trigger protective responses. These responses include the secretion of cytokines that increase the resistance of infected cells as well as the release of chemokines that recruit immune cells to dead cells, thus limiting microbe spreading.

Viral dsRNA participates in virus-infected cell apoptosis, but the signaling pathway involved remains unclear. Salaun et al. [13] showed that synthetic dsRNA induce apoptosis of human breast cancer cells in a TLR3-dependent manner. This mechanism involves the molecular adaptor Toll/IL-1R domain-containing adapter inducing IFN- and type I IFN autocrine signaling, but occurs independently of the dsRNA-activated kinase. The role of TLRs expressed by tumor cells in the evasion of immune surveillance was demonstrated in animal experiments [14]. These results showed that TLRs stimulation may lead to tumor progression and that there are now means to specifically reverse this unwanted effect.

The purpose of the present study was to investigate the expression of TLR3, TLR4 and TLR9 in breast cancer as well as its relation to distant metastasis. To address these questions, we analyzed the protein levels of TLR3, TLR4 and TLR9 by tissue arrays technology (TA) and immunohistochemical techniques, and their mRNA levels by real time-PCR.

## Methods

### Patients

This study included 74 women with histologically confirmed early breast cancer confirmed and treated between 1990 and 2003. We selected patients with the following inclusion criteria: invasive ductal carcinoma, in cases of non-recurrence, patients had been followed-up for a minimum of 5 years of follow-up. The exclusion criteria were the following: metastatic disease at presentation, prior history of any kind of malignant tumor, bilateral breast cancer at presentation, any type of neoadjuvant therapy, development of loco-regional recurrence during the follow-up period, development of a second primary cancer, and absence of sufficient tissue in paraffin blocks. From a total of 1,264 patients fulfilling these criteria, we randomly selected a sample size of 74 patients in accordance divided them into two different groups of similar size and stratified each group with regard to the development of metastasis. Patients and tumor characteristics are listed in Table 1. Patients were treated according to approved guidelines at our institution. The study adhered to national regulations and was approved by our institutions Ethics and Investigation Committee. Tissue samples were obtained prior informed consent from the patients. All patients were followed for distant metastasis status by clinical and biological studies every 3 months for the first 2 years and then yearly. Radiological studies were performed yearly, or when considered necessary. The end-point was distant metastatic relapse. The median follow-up period was 85 months in patients without metastases, and 46 months in patients with metastases.

**Table 1:** Basal characteristics of 74 patients with invasive carcinoma of the breast.

CHARACTERISTICS	WITHOUT RECURRENCE N(%)	WITH RECURRENCE N(%)
**Age (years)**		

<57	14 (48.2)	29 (64.4)

>57	15 (51.8)	16 (35.6)

**Menopausal status**		

Premenopausal	11 (37.9)	11 (24.4)

Postmenopausal	18 (62.1)	34 (75.6)

**Tumoral size**		

T1	14 (48.2)	15 (33.3)

T2	15 (51.8)	30 (66.7)

**Nodal status**		

N-	14 (48.2)	22 (48.8)

N+	15 (51.8)	23 (51.2)

**Histological grade**		

Well dif	11 (37.9)	10 (22.2)

Mod dif	15 (51.7)	20 (44.4)

Poorly dif	3 (10.4)	15 (33.4)

**Nottingham pronostic index**		

<3.4	10 (34.4)	12 (26.7)

3.4-5.4	16(55.1)	21 (46.6)

>5.4	3 (10.5)	12 (26.7)

**Estrogen receptors**		

Negative	10 (34.4)	28 (62.8)

Positive	19 (65.6)	17 (37.8)

**Progesterone receptors**		

Negative	11 (37.9)	32 (71,1)

Positive	18 (62.1)	13 (28,9)

**Desmoplasia**		

Negative	11 (37.9)	11 (24.4)

Positive	18 (62.1)	34 (75.5)

**Peritumoral inflammation**		

No	15 (51.7)	20 (44.4)

Mild	13 (44.8)	24 (53.3)

Intense	1 (3.5)	1 (2.3)

**Tumor progress**		

Expansive	16 (55.1)	17 (37.7)

Infiltrating	13 (44.9)	28 (62.3)

**Mitosis**		

<10	16 (55.1)	18 (40)

>10	13 (44.9)	27 (60)

**Tumoral necrosis**		

No	26 (89.6)	37 (82.2)

Focal	2 (6.9)	7 (15.5)

Extense	1 (3.5)	1 (2.3)

### Tissue array immunohistochemistry and analysis

Breast carcinoma tissue samples were obtained at the time of surgery. All specimens were routinely fixed in 10% neutral buffered formalin and stored in paraffin at room temperature for a period of four months to five years before further testing. Histopathological representative tumor areas were defined on haematoxylin and eosin-stained sections and marked on the slide. Tumour tissue array (TA) blocks were obtained by punching a tissue cylinder (core) with a diameter of 1.5 mm through a histological representative area of each 'donor' tumor block, which was then inserted into an empty 'recipient' TA paraffin block using a manual tissue arrayer (Beecher Instruments, Sun Prairie, Winconsin, USA) as described elsewhere [15]. Collection of tissue cores was carried out under highly controlled conditions. Two cores were employed for each case.

Four composite high-density TA blocks were designed, and serial 5-μm sections were consecutively cut with a microtome (Leica Microsystems GmbH, Wetzlar, Germany) and transferred to adhesive-coated slides. One section from each TA block was stained with haematoxylin and eosin, and these slides were then reviewed to confirm that the sample was representative of the original tumor. Immunohistochemistry was done on these sections of TA fixed in 10% buffered formalin and embedded in paraffin using a TechMate TM50 autostainer (Dako, Glostrup, Denmark). Monoclonal antibodies for TLR3 (TLR3.7; ref: sc-32232), TLR4 (H-80; sc-10741), and TLR9 (H-100; sc-25468) were obtained from Santa Cruz Biotechnology (California, USA). The dilution for each antibody was established based on negative and positive controls (1/50 for TLR3, 1/100 for TLR4 and TLR9).

Tissue sections were deparaffinized in xylene, and then rehydrated in graded concentrations of ethyl alcohol (100%, 96%, 80%, 70% and water). To enhance antigen retrieval for the three antibodies, TA sections were microwave-treated (H2800 Microwave Processor, EBSciences, East Granby, Connecticut, USA) in citrate buffer, (Target Retrieval Solution, Dako), with high pH (pH9) for TLR3 and low pH (pH6) for TLR4 and 9, at 99°C for 16 min. Endogenous peroxidase activity was blocked by incubating the slides in peroxidase-blocking solution (Dako) for 5 min. The EnVision Detection Kit (Dako) was used as the staining detection system. Sections were counterstained with haematoxylin, dehydrated with ethanol, and permanently coverslipped.

The location of immunoreactivity, percentage of stained cells, and intensity were determined for each antibody preparation. All the cases were semiquantified for each protein-stained area. An image analysis system using the Olympus BX51 microscope and analysis soft (analySIS^®^, Soft imaging system, Münster, Germany) was employed as follows: tumor sections were stained with antibodies according to the method explained above and counterstained with haematoxylin. There were different optical thresholds for both stains. Each core was scanned with a 400× power objective in two fields per core. Fields were selected on the basis of protein-stained areas. The computer program selects and traces a line around antibody-stained areas (red spots) for higher optical threshold. The remaining non-stained areas (haematoxylin-stained tissue with lower optical threshold) appear as a blue background. Each field has an area ratio of stained (red) versus non-stained areas (blue). A final area ratio was obtained after averaging two fields. To evaluate immunostaining intensity we used a numeric score ranging from 0 to 3, reflecting the intensity as follows: 0, no staining; 1, weak staining; 2, moderate staining; and 3, intense staining. Using an Excel spreadsheet, the mean score was obtained by multiplying the intensity score (I) by the percentage of stained cells [16] and the results were added together (total score: I × PC). This overall score was then averaged with the number of cores that were done for each patient. If there was no tumor in a particular core, then no score was given. In addition, for each tumor, the mean score of two core biopsies was calculated.

### Western blot

Samples were separated by SDS-PAGE using 10% polyacrylamide gels and run at constant 120 V (Mini-Protean^® ^Tetra Electrophoresis System, Bio-Rad, Hercules, USA). The tetraprotean transference kit was used to electrotransfer proteins to nitrocellulose membranes at 160 mA for 1 h in transfer buffer (0.248 M Tris pH 8.8, 1.92 M glycine and 20% methanol). The nitrocellulose filters containing the transferred proteins were blocked by rocking for 1 h in Tris-buffered saline (TBS) contained 1% skimmed milk and rinsed 3 tines in TBS. The filters were incubated for 2 h at room temperature with one of these monoclonal antibodies: anti-TLR3 (ref: sc-32232), anti-TLR4 (sc-10741), anti-TLR9 (sc-25468) (Santa Cruz Biotechnology, California, USA) diluted in TBS containing 1% skimmed milk. The blots were then washed with TBS, incubated with protein A peroxidase and the reactive protein bands were visualized by chemiluminiscence (Pierce ECL Western Blotting Substrate, Rockford, USA).

### Real-time PCR

Total RNA was isolated from breast tissue using the RNeasy Mini kit (Quiagen, Hilden, Germany), including DNase treatment. The integrity of the eluted total RNA was checked by agarose gel electrophoresis and the RNA concentration was determined spectrophotometrically. First strand cDNA was made using the High Capacity cDNA Reverse Transcrition kit (Applied Byosystems, Cheshire, UK) following the manufacturer's instructions. The reverse transcription step was carried using the following program: 25°C for 10 min, 37°C for 120 min and 85°C for 5 sec. The expression levels of TLR3, TLR4, TLR9 and β-actin were assessed by real-time PCR using ABI Prism 7900 HT thermocycler (Applied Biosystems, Cheshire, UK) and the Fast SYBR Green Master Mix (Applied Biosystems, Cheshire, UK) with the following cycling conditions: 95°C for 20 sec, 40 cycles of 95°C for 1 sec and 60°C for 20 sec. The primers used were 5'-TAGCAGTCATCCAACAGAATCAT-3' (forward) and 5'-AATCTTCTGAGTTGATTATGGGTAA-3' (reverse) for TLR3, 5'-ACTCCCTCCAGGTTCTTGATTAC-3' (forward) and 5'-CGGGAATAAAGTCTCTGTAGTGA-3' (reverse) for TLR4, 5'-CTTCCCTGTAGCTGCTGTCC-3' (forward) and 5'-CCTGCACCAGGAGAGACAG-3' (reverse) for TLR9 and 5'-GGCACCCAGCACAATGAAG-3' (forward) and 5'-CCGATCCACACGGAGTACTTG-3' (reverse) for β-actin. PCR products were separated on 2% agarose gels containing ethidium bromide (0.5 μg/ml).

### Data analysis and statistical methods

Differences in percentages were calculated with the chi-square test. Immunostaining score values for each protein were expressed as median (range). A comparison of group immunostaining values was made with the Mann-Whitney or Kruskal-Wallis tests. For metastasis-free survival analysis we used the Cox's univariate method. Cox's regression model was used to examine interactions of different prognostic factors in a multivariate analysis. The SPSS 17.0 program was used for all calculations.

## Results

**Figure 1:**
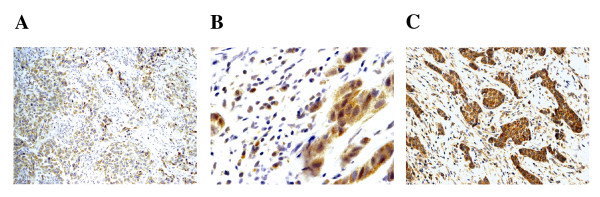
**Examples of immunostaining for TLRs analyzed: A) TLR3 positive staining for tumor cell**. 200× B) TLR4 positive staining for tumor cell and mononuclear inflammatory cells. 400×, and C) TLR9 positive staining for tumor cell and fibroblast-like cells. 200×.

In the present study, we investigated the expression levels of TLR3, TLR4 and TLR9 in tumors from 74 women with ductal invasive breast cancer. Figure 1 shows examples of immunostaining for these proteins. TLRs showed an intracellular location pattern, but TLR3 also was localized in the cell surface. Positive staining was generally found in cancer cells but also in some stromal cells (fibroblast-like cells as well as in mononuclear inflammatory cells -MICs-). Table 2 sumarizes the percentages of each TLR staining in each cellular type. In tumors, cancer cells exhibited high expressions, especially of TLR4 and TLR9. Nevertheless, a significant percentage of tumors also showed expression **levels **of TLR4 by MICs (21.6%) and of TLR9 by fibroblast-like cells (57.5%).

**Table 2:** TLRs expression in 74 cases of breast cancer.

Factor	Tumoral cells	Fibroblast	MICs
	
	**n**^**o**^**total cases** **n**^**o**^**positive cases (%)**	**n**^**o**^**total cases** **n**^**o**^**positive cases (%)**	**n**^**o**^**total cases** **n**^**o**^**positive cases (%)**
**TLR3**	52 (79)	2 (3)	2 (3)

**TLR4**	70 (95)	6 (8)	16 (22)

**TLR9**	73 (100)	42 (58)	5 (7)

Abbreviations: MICs: mononuclear inflammatory cells.

**Figure 2:**
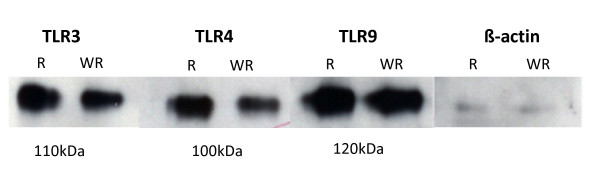
**Representative western blots of immunoreactive toll-like receptors (TLRs)**. Twenty micrograms of whole cell extract from human breast carcinoma with (R) and without recurrence (WR) were subject to 10% SDS-PAGE and transferred into a nitrocellulose membrane and then immunolabelled with TLR3, TLR4, TLR9 and β-actin (used as loading control) antiserum.

The presence of the TLR gene products was confirmed by western blot in breast tumor samples (Figure 2). The results clearly showed immunoreactive bands corresponding to TLR3, TLR4 and TLR9. As shown in Figure 2 anti-TLR3, anti-TLR4 and anti-TLR9 recognized bands of approximately 110, 100 and 120 KDa, respectively.

**Figure 3:**
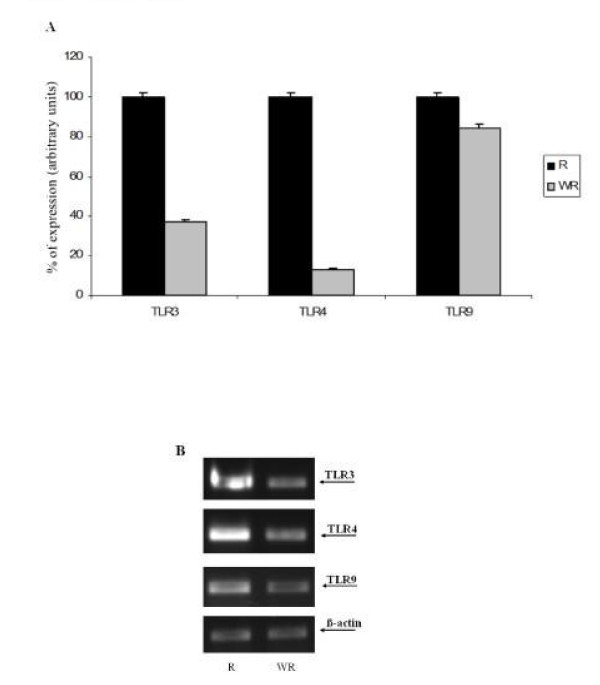
**TLR3, 4 and 9 gene expression measured by semiquantitative real time PCR in breast carcinomas**. Upper panel (A), shows the percentage of TLRs expression in recurrence samples and without recurrence ones (WR). Lower panel,(B) shows the electrophoresis bands after real time-PCR performed on equal amounts of cDNA from each sample. The housekeeping used was β-actin. Data represent the mean SD of three independent experiments.

Figure 3 shows the results of real time PCR. The percentage of TLR cDNA expression in samples obtained from breast cancer patients with recurrence and from breast cancer patients without recurrence are compared in the upper panel whereas the electrophoresis analysis is shown in the lower panel. We found elevated TLR expression levels in tissue samples from patients with recurrence relative to samples from patients without recurrence.

**Figure 4:**
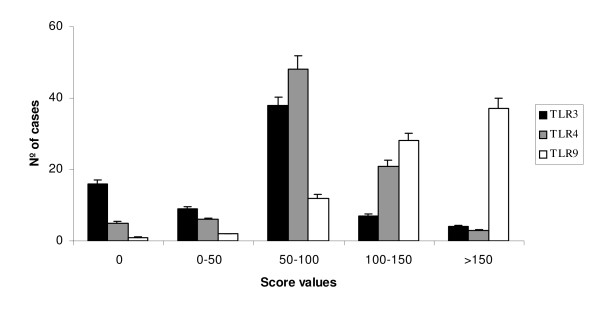
**the immunostaining score values for TLR3,4 and 9 in breast carcinomas**.

Figure 4 shows the immunostaining score values, which ranged widely for each TLR. We also evaluated the possible relationship between the TLRs expressions and clinicopathological factors of breast carcinomas including menopausal status, tumor size, nodal status, tumor stage, histological grade, estrogen and progesterone receptors, tumor advancing edge, peri-tumor inflammation and tumor necrosis as summarized in Table 3. Both TLR3 and TLR4 expressions were significantly and positively associates with tumor size. A significant association between TLR3 or TLR9 expression score and tumor stage was also found. In addition, when compared with tumors from premenopausal women, we found that tumors from postmenopausal women had significantly higher TLR3 and TLR4 score values (Table 3).

**Table 3:** stadistical analysis between TLRs expressions and clinicopathological factors in women with breast carcinomas.

Characteristics	TLR3		TLR4		TLR9	
	**median (range)**	**p**	**median (range)**	**p**	**median (range)**	**p**

**Menopausal Status**						

Premenopausal	51.4 (0-158)	**0.048**	57.37 (0-160)	**0.006**	138.7 (46-268)	0.319

Postmenopausal	65.9 (0-166)		72.33 (0-158)		153 (37-272)	

**Tumoral size**						

T1	49.7 (0-166)	**0.005**	62.7 (0-160)	**0.039**	140.2 (37-272)	0.269

T2	69.2 (0-162)		71.2 (38-158)		151.4 (46-268)	

**Nodal status**						

N-	60.5 (0-162)	0.717	68 (0-143)	0.725	147.5 (38-246)	0.724

N+	60.5 (0-166)		68.7 (36-160)		153 (46-272)	

**Estrogen receptors**						

Negative	63.7 (0-166)	0.923	67.1 (0-159)	0.436	150.5 (46-272)	0.904

Positive	59.9 (0-162)		69.4 (0-156.2)		148 (37-268)	

**Progesterone receptors**						

Negative	67.3 (0-166)	0.101	70 (0-158)	0.396	150.3 (46-272)	0.251

Positive	57.1 (0-158)		62.8 (38-135)		137.4 (37-268)	

**Tumoral progess**						

Expansive	62.7 (0-162)	0.863	71.6 (0-160)	0.247	151.8 (65-272)	0.167

Infiltrating	59.9 (0-166)		62.1 (0-156)		143.1 (38-268)	

**Stage**						

I	46 (0-70)	**0.024**	62.8 (0-135)	0.175	136.9 (37-180)	**0.035**

II	62.4 (0-162)		69.3 (38-160)		155.9 (46-268)	

III	72.3 (0-166)		69 (36-158)		147.5 (60-272)	

**SBR**						

SBRI	57.1 (0-102)	0.671	62.8 (0-135)	0.201	137.9 (38-272)	0.218

SBRII	57.9 (0-166)		66.5 (0-160)		148.4 (60-246)	

SBRIII	64.3 (0-158)		96.2 (0-158)		161.7 (46-268)	

**Peritumoral inflamation**						

No	57.9 (0.162)	0.815	67.5 (0-156)	0.815	141.7 (37-272)	0.191

Mild	61 (0-166)		69.4 (0-160)		158,2 (46-268)	

Intense	60 (50-70)		88.4 (62-114)		137.7 (115-159)	

**Tumoral necrosis**						

No	57.5 (0-166)	0.359	68 (0-158)	0.184	147.5 (38-272)	0.095

Focal	69.7 (0-162)		64.4 (46-125)		151.1 (119-173)	

Extense	71.4 (29-113)		133.2 (106-160)		214.6 (183-246)	

**Figure 5:**
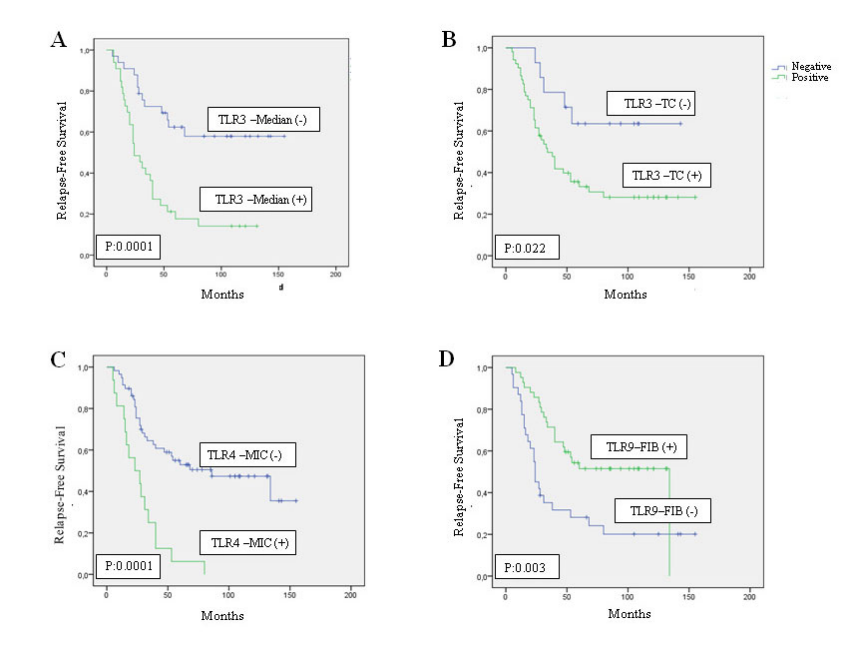
**A) Probability of biochemical recurrence as function of TLR3 median (p:0.0001), B) probability of biochemical recurrence as function of TLR3 expression by tumor cells (p: 0.022)**. C) probability of biochemical recurrence as function of TLR4 in monocites (p: 0001) and D) probability of biochemical recurrence as function of TLR9 expression by intratumor fibroblasts (p: 0.003).

We also analyzed the possible relationship between TLRs immunostaining values and distant relapse-free survival. Univariate analysis (Figure 4) demonstrated that high score values for TLR3 expression, TLR3 expression by tumoral cells, or TLR4 expression by MICs, were significantly associated with a great rate of distant metastasis. However, TLR9 expression by fibroblast-like cells was significantly associated with low rate of distant metastases (Table 4 and Figure 5). Multivariate analysis with a Cox model demonstrated that tumour stage and progesterone receptor-status were significantly and independently associated with relapse-free survival in patients with breast cancer. However, this same analysis also demonstrated that TLRs expressions are significantly associated with prognosis (Table 4).

**Table 4:** Cox's univariate (HR) and multivariate (RR) analysis of the relationship between TLRs expression and relapse-free survival.

Factor	Number of patients	Event frequency	HR (95% CI)	RR (95% CI)
**TLR3**				

Score<median>median	33/33	13/28	3.4 (1.8-6.7)**	2.6 (1-3-5.1)**

CT (-) vs (+)	14/52	may-36	2.8 (1.1-7.2)*	2.5 (0-9-6.5)*

**TLR4**				

MIC (-) vs (+)	58/16	29/16	3.7 (2-7)**	3.5 (1.8-6.8)**

**TLR9**				

F (-) vs (+)	31/42	24/21	0.4 (0.2-0.7)**	0.3 (0.3-0.6)**

**Tumoral stage II vs III**	38/20	20/17	1.9 (1.3-3) **	2.8 (1.2-6.5)*

**Progesterone receptors positive vs negative**	42/30	31/13	0.4 (0.2-0.8)*	0.3 (0.1-0.7)**

*p < 0.005; **p < 0.05

## Discussion

This study analyzes tumor expression as well as the prognostic significance of TLRs in breast cancer. The results demonstrated an association TLR3, TLR4 and TLR9 expression and distant metastasis.

TLRs expression has been described in different human tumors [13,17-28]. Receptor-deficient mice were found to be protected from or develop less inducible tumors in experimental models [22,29]. Cancer cells activated by TLR signals may release cytokines and chemokines that in turn may recruit immune cells and stimulate them to release further cytokines and chemokines. This process results in a cytokine profile that is associated with immune tolerance, cancer progression and propagation of the tumor microenvironment [30]. Recent evidences also show that functional TLRs may play an important role in tumor progression by activating the production of interleukins, tumor-necrosis factor-alpha (TNF-α), nuclear factor-kappaBeta (NF-kappaB) and metalloproteases [31,32]. Likewise, activation of tumor cell TLRs not only promotes tumor cell proliferation and resistance to apoptosis, but also enhances tumor cell invasion and metastasis by regulating metalloproteases and integrins [33-35]. Although expression of these factors was generally associated with an adverse prognosis, the expression pattern of TLRs in human breast cancer tissues is largely unknown. Our results show high expression of TLR3, TLR4 and TLR9 by breast cancer cells though.

Our results showed that TLR3 expression is associated with high probability of metastasis, which is in agreement with previous studies indicating that TLR3 expression is related to tumoral aggressiveness [13,36-39]. Therefore, TLR3 may represent a good therapeutic target in breast cancer. In this sense, there are studies showing a variable antineoplastic effect caused by a blockade of TLR3 [40,41].

The TLR4 expression by MICs and/or TLR9 by fibroblast-like cells is another interesting finding of our study. TLR4 expression by MICs was associated with an increased incidence of metastasis, whereas TLR9 expression by fibroblast-like cells was associated a low metastasis-rate. These findings support data from other authors on the importance of the tumor stromal cells in tumor behavior. The role of stromal cells has been attributed to the release by them of various extracellular matrix proteins, growth factors, proteases and other factors that act as signal transducers for tumor progression [42-52]. Therefore, our results also suggest the existence of different phenotypes of stromal cells that influence prognosis depending on their TLR expression pattern.

TLR4 recognizes several bindings which in turn activate transcription factors, resulting in the expression and release of cytokines such as interleukin-1, interleukin-6 and interleukin-8 [53]. Consequently, considering the fact that the TLR4 expression by MICs is increased in breast cancer cases with recurrence, our results suggest that the use of TLR4 agonists may become a useful anticancer strategy [18,54,55].

Our data also suggest that TLR9 may help to identify one population of fibroblast-like cells associated with good prognosis. Although the biological significance of TLR9 expression by stromal fibroblast-like cells is currently unknown, there are data pointing to a protective role of this receptor against tumoral progression. Indeed, it was demonstrated that stimulation of TLR-9 activates human plasmacytoid dendritic cells and B cells, thus inducing potent innate immune responses in preclinical tumor models as well as in patients [56]. It is therefore understandable that using bindings of nucleic acid-sensing TLR9 as a pharmacological intervention in various diseases is gaining in interest.

## Conclusions

Our results show that TLR expression have prognostic significance and suggest that these markers may represent new therapeutic targets in breast cancer. Further studies on the TLRs expression in tumor context may help to better understand the process that links inflammation and cancer, as well as to assess the biological and clinical importance of the interplay between tumor and stroma in breast cancer.

## Competing interests

The authors declare that they have no competing interests.

## Authors' contributions

guarantor of integrity of the entire study- GRS and VF; study concepts and design- GRS, ML and VF; literature research- GRS, LM and VF; clinical studies- GRS, ML, GL, GLO and GJ; experimental studies/data analysis- GRS, ML, GL, GLO and GJ; statistical analysis DCJ; manuscript preparation- GRS and VF; manuscript editing- GRS and VF. All authors read and approved the final manuscript.

## Pre-publication history

The pre-publication history for this paper can be accessed here:

http://www.biomedcentral.com/1471-2407/10/665/prepub
